# Endoscopic cyanoacrylate injection with gel immersion method improved the visual field in profuse gastric variceal hemorrhage

**DOI:** 10.1055/a-2068-7850

**Published:** 2023-04-26

**Authors:** Daisuke Orita, Tsukasa Ishida, Yoshihiro Okabe, Yuichi Hirata, Michitaka Kohashi, Takuya Mimura, Akihiko Nishizawa

**Affiliations:** 1 Department of Gastroenterology, Kakogawa City Central Hospital, Kakogawa, Japan; 2 Department of Gastroenterology, Akashi Medical Center, Akashi, Japan

Endoscopic cyanoacrylate injection is recommended for gastric variceal hemorrhage (GVH) as a means of achieving urgent endoscopic hemostasis 1 2 . However, it can sometimes be technically difficult to secure the visual field because GVH produces a large amount of blood and bloody clots immediately fill the fundus. Gel immersion endoscopy (GIE) is useful for identification of the gastrointestinal bleeding point as it offers a secure visual field 3 4 5 . We report a case of successful hemostasis for GVH using cyanoacrylate injection under GIE ( Video 1 ).

**Video 1** Endoscopic hemostasis for gastric variceal hemorrhage was achieved by cyanoacrylate injection under gel immersion endoscopy.


A 70-year-old man with liver cirrhosis due to nonalcoholic fatty liver disease presented hematemesis. Emergency endoscopy identified active bleeding from ruptured gastric varices located in the fundus ( Fig. 1 ). We immediately attempted endoscopic hemostasis, but the visual field became poor due to the large amount of blood, and gas insufflation and water immersion methods were ineffective in improving the visual field ( Fig. 2 a ). Electrolyte-free gel (Viscoclear; Otsuka Pharmaceutical Factory, Tokushima, Japan) was therefore injected through the accessory channel using a waterjet pump (OFP; Olympus, Tokyo, Japan) with an auxiliary injection cap (BioShield irrigator; US Endoscopy, Mentor, Ohio, USA) ( Fig. 3 ). Continuous gel injection gradually improved the visual field ( Fig. 2 b ). Even with active bleeding, it was possible to identify the bleeding point because gel and blood took time to merge. Endoscopic cyanoacrylate injection was completed with gel immersion. A total of 500 mL of this gel was used, and a total of 4 mL of 62.5 % cyanoacrylate with lipiodol was injected into the varices over five tries, resulting in complete hemostasis. Cyanoacrylate with lipiodol pooling in varices was confirmed by computed tomography ( Fig. 4 ).

**Fig. 1:**
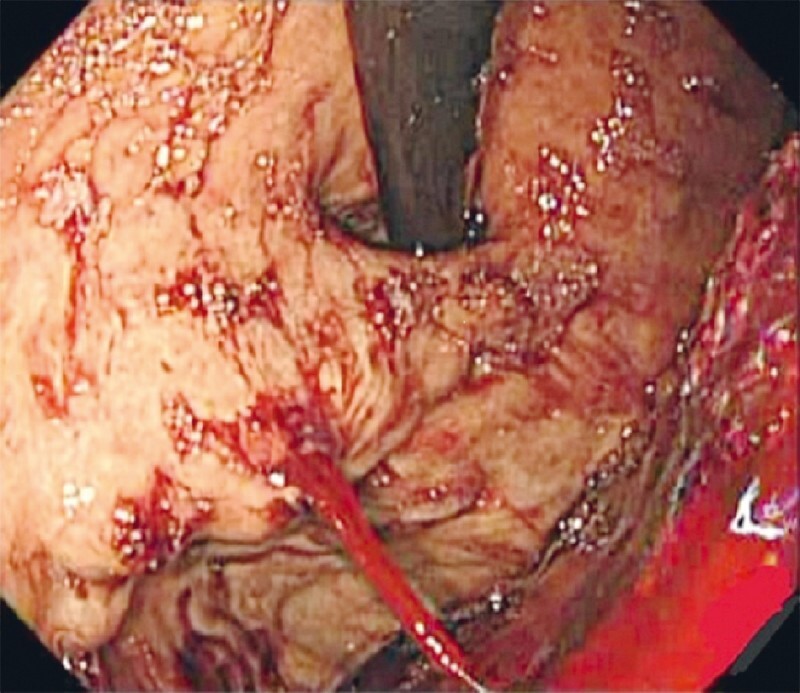
Active bleeding from gastric varices located in the fundus.

**Fig. 2:**
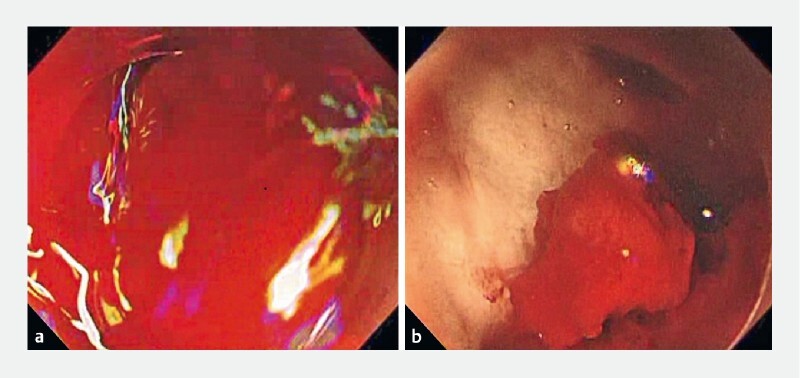
The difference in endoscopic visual fields between water and gel immersion methods. **a** The water immersion method was ineffective in improving the visual field. **b** The gel immersion method improved the visual field and the bleeding point could be identified.

**Fig. 3:**
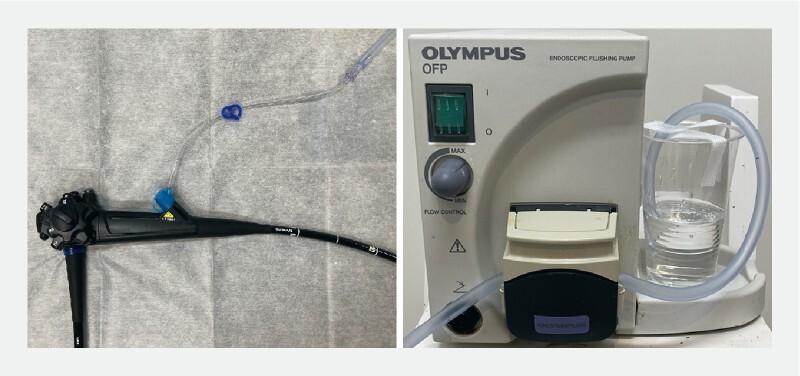
Endoscopic settings for gel immersion endoscopy. Gel immersion endoscopy was by therapeutic endoscope with waterjet function (GIF-290T; Olympus, Tokyo, Japan). A plastic cup containing the viscous gel was connected to the waterjet system via an auxiliary injection cap.

**Fig. 4:**
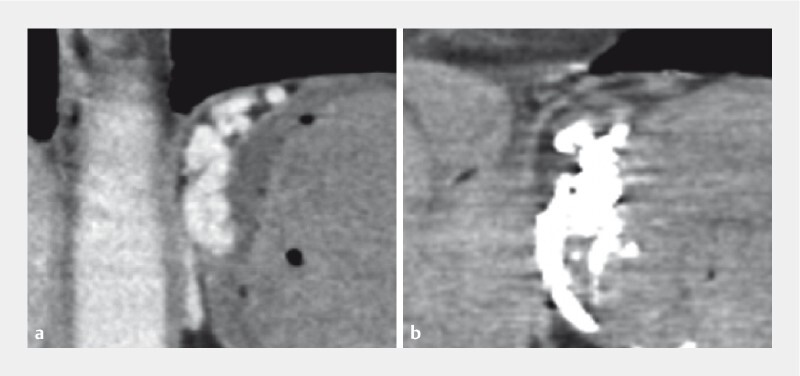
Computed tomography scan evaluation after cyanoacrylate injection. The scan showed good pooling of cyanoacrylate with lipiodol after injection. **a** Before injection. **b** After injection.

To our knowledge, this is the first report of the successful use of endoscopic cyanoacrylate injection with GIE for active GVH. GIE was able to maintain a good visual field, even in a case of massive bleeding.

Endoscopy_UCTN_Code_CCL_1AB_2AC_3AG

## Notes

Endoscopy E-Videos

https://eref.thieme.de/e-videos
*E-Videos* is an open access online section of the journal *Endoscopy* , reporting on interesting cases and new techniques in gastroenterological endoscopy. All papers include a high-quality video and are published with a Creative Commons CC-BY license. Endoscopy E-Videos qualify for HINARI discounts and waivers and eligibility is automatically checked during the submission process. We grant 100% waivers to articles whose corresponding authors are based in Group A countries and 50% waivers to those who are based in Group B countries as classified by Research4Life (see: https://www.research4life.org/access/eligibility/ ). This section has its own submission website at https://mc.manuscriptcentral.com/e-videos

## References

[1] Iwase H Maeda O Shimada M Endoscopic ablation with cyanoacrylate glue for isolated gastric variceal bleeding Gastrointest Endosc 2001 53 585 592 11323583 10.1067/mge.2001.113921

[2] Chirapongsathorn S Manatsathit W Farrell A Safety and efficacy of endoscopic cyanoacrylate injection in the management of gastric varices: a systematic review and meta-analysis JGH Open 2021 5 1047 1055 34584974 10.1002/jgh3.12629 PMC8454477

[3] Yano T Nemoto D Ono K Gel immersion endoscopy: a novel method to secure the visual field during endoscopy in bleeding patients (with videos) Gastrointest Endosc 2016 83 809 811 26463338 10.1016/j.gie.2015.09.048

[4] Kobayashi Y Ando K Sasaki T Usefulness of endoscopic band ligation with gel immersion endoscopy for colonic diverticular bleeding and hemorrhoidal bleeding Endoscopy 2022 54 384 385 34374049 10.1055/a-1550-1913

[5] Sekiguchi H Yano T Tokoro S Low-pressure endoscopy using the gel immersion method facilitates endoscopic variceal ligation of ruptured esophageal varices Endoscopy 2022 54 828 829 34535032 10.1055/a-1559-2120

